# DiMeX: A Text Mining System for Mutation-Disease Association Extraction

**DOI:** 10.1371/journal.pone.0152725

**Published:** 2016-04-13

**Authors:** A. S. M. Ashique Mahmood, Tsung-Jung Wu, Raja Mazumder, K. Vijay-Shanker

**Affiliations:** 1 Department of Computer and Information Sciences, University of Delaware, Newark, Delaware, United States of America; 2 Department of Biochemistry and Molecular Medicine, George Washington University, Washington, District of Columbia, United States of America; 3 McCormick Genomic and Proteomic Center, George Washington University, Washington, District of Columbia, United States of America; Garvan Institute of Medical Research, AUSTRALIA

## Abstract

The number of published articles describing associations between mutations and diseases is increasing at a fast pace. There is a pressing need to gather such mutation-disease associations into public knowledge bases, but manual curation slows down the growth of such databases. We have addressed this problem by developing a text-mining system (DiMeX) to extract mutation to disease associations from publication abstracts. DiMeX consists of a series of natural language processing modules that preprocess input text and apply syntactic and semantic patterns to extract mutation-disease associations. DiMeX achieves high precision and recall with F-scores of 0.88, 0.91 and 0.89 when evaluated on three different datasets for mutation-disease associations. DiMeX includes a separate component that extracts mutation mentions in text and associates them with genes. This component has been also evaluated on different datasets and shown to achieve state-of-the-art performance. The results indicate that our system outperforms the existing mutation-disease association tools, addressing the low precision problems suffered by most approaches. DiMeX was applied on a large set of abstracts from Medline to extract mutation-disease associations, as well as other relevant information including patient/cohort size and population data. The results are stored in a database that can be queried and downloaded at http://biotm.cis.udel.edu/dimex/. We conclude that this high-throughput text-mining approach has the potential to significantly assist researchers and curators to enrich mutation databases.

## Introduction

Rapidly evolving sequencing technologies [1,2] have led to a dramatic rise in the number of published articles reporting associations between genomic variations and diseases. There is an estimate that over 10,000 articles are published each year mentioning such associations [3]. Manually collecting this information is both expensive and time consuming. Uniprot [4], COSMIC [5], BioMuta [6], OMIM [7], HGMD [8], UMD [9], HGVbaseG2P [10], MutDB [11], dbSNP [12], PharmGKB [13], ClinVar [14] and InSiGHT [15] are examples of repositories that house mutations and related disease and phenotype information laboriously manually curated from the literature. Manual curation cannot keep up with the new information being published every year.

To assist this manual curation, several text-mining (TM) efforts [16–27] have been attempted. However, most of these efforts are limited to identifying mutation mentions only. The majority utilize regular expressions to detect mutations, although there are some, like tmVar [28] and VTag [29], that use conditional random fields (CRFs), and SETH [30], which implements an Extended Backus-Naur Form (EBNF) grammar. Only a few of these efforts extend the mutation detection method to associate the mutation with a disease phenotype. Most of these are search based TM tools that do not employ automatic extraction of the mutation-disease relationships expressed in articles. PolySearch [31] is such a search-based TM tool that infers relationships between mutations and diseases based on their frequency of co-occurrence in Medline abstracts. The work reported in Schenck et al. [32] combines existing TM methods into a workflow to associate mutations with cancers from text with high precision but low recall. To the best of our knowledge, EMU [16] is the only TM method that extracts mutations from abstracts, attaches them to associated genes, and finally couples them with associated diseases.

In this study, we propose a novel text mining approach that extracts mutations from Medline abstracts and identifies their association with diseases. In contrast to co-occurrence based systems that associate mutations with diseases on the basis of their co-occurrence in the same abstract, we use information extraction techniques in addition to co-occurrence to capture the relationship. Co-occurrence based approaches look at multiple articles for frequencies of co-occurrence in order to derive a confidence for an association that is text mined. In contrast, we extract information from a single abstract and use sentence structure together with other textual features to base the confidence of the extraction. An association between a mutation and a disease can be expressed in various ways in text. In some cases, one part of an association, e.g., the disease, might be mentioned in a different sentence than the associated mutation. Under the hypothesis that this should be inferable from the nearby context, we use textual clues to find it and make the linkage.

Our system is comprised of a series of natural language processing (NLP) modules including syntactic preprocessing of input text, detecting different types of mutations, a novel algorithm to associate mutations with genes, an information extraction (IE) module to apply lexical and semantic patterns to extract associations between mutations and diseases, and additional rules to infer associations beyond patterns. We also extract additional information, including the number of patients, the race or nationality of the patients and whether the mutation is associated with the outcome (e.g., overall survival) of the disease or the efficacy of a drug therapy for the disease.

We have evaluated our system on two different annotated sets of data. One is from the BioMuta [6] project, which pertains to human cancers, and the other, used for evaluating the mutation-disease association system of Doughty et al. [16], pertains to only prostate and breast cancers. We achieved precision of 0.87, recall of 0.89 and F-measure of 0.88 on the BioMuta set. For the latter set, we achieved precision of up to 0.95, recall of 0.88, and F-measure of 0.91, which indicates that our system outperforms an existing tool described in Doughty et al. [16] for mutation-disease association that achieves precision of up to 0.76, recall of 0.75 and F-measure of 0.75 on the same dataset. The component for detecting mutation mentions also achieved similar performance to the current state-of-the-art system tmVar [28] (F-measures of 0.94, 0.94 and 0.91) on three different corpora: Mutation Finder [20], Variome [33] and tmVar [28]. We also show that our mutation to gene association module achieves F-measures of up to 0.93 on the BioMuta dataset and 0.94 on the prostate and breast cancer datasets, while the system of Doughty et al. [16] achieves 0.76 and 0.68 on the latter datasets, respectively.

We applied our system on roughly 10,000 abstracts. The extraction results are stored in a database, which can be queried and downloaded for further analysis. The database is accessible via a simple interface that we developed (http://biotm.cis.udel.edu/dimex/). Some sample queries showing the possible uses of the system are presented in the Results section of this article. We conducted an evaluation of a sample of extractions from the database and found it to be consistent with our evaluations mentioned above. This exercise illustrates the scalability and robustness of DiMeX.

## Materials and Methods

**Fig 1:**
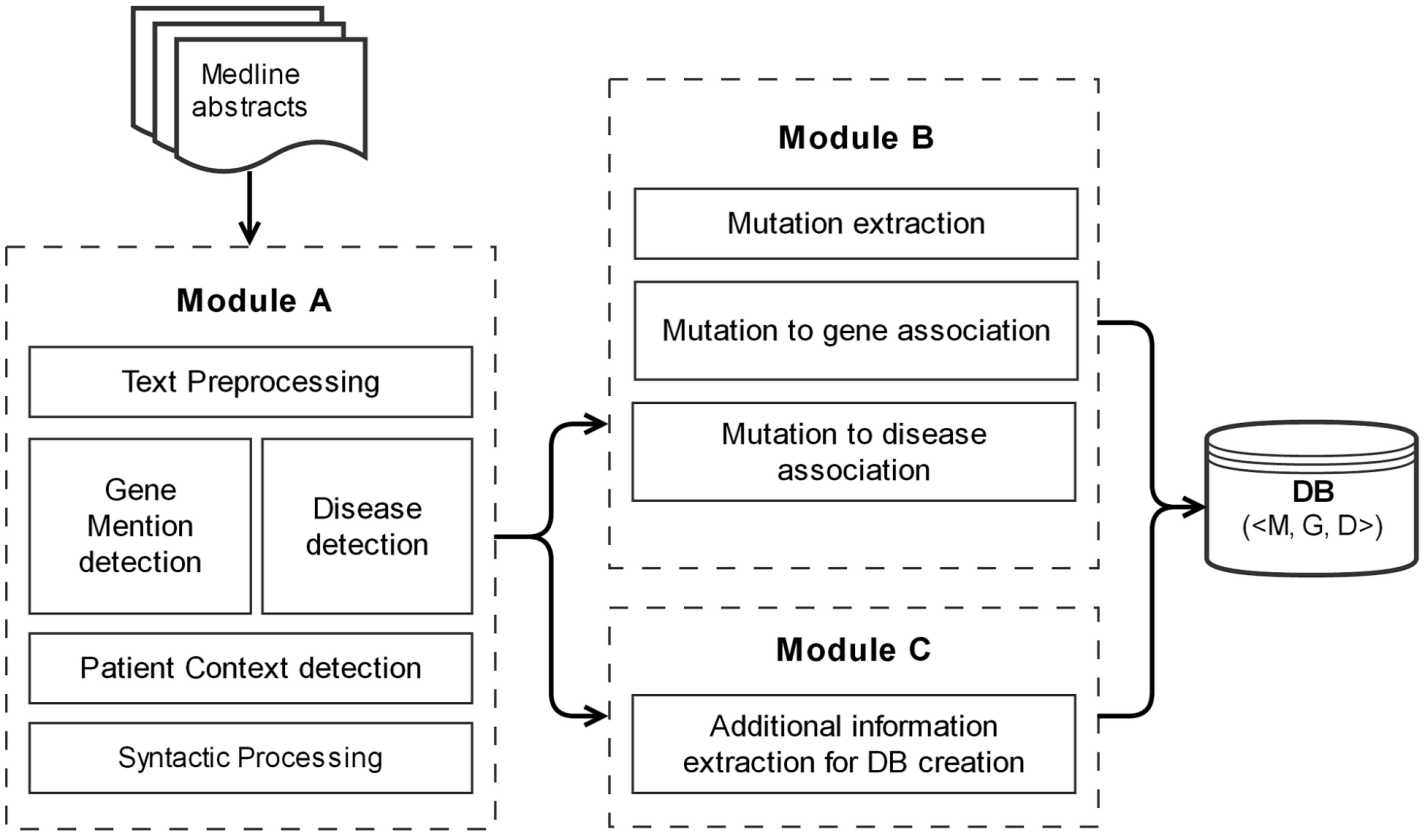
Schematic diagram of DiMeX.

The schematic diagram of the overall architecture of the system is shown in Fig 1. The system is divided into three main modules, namely A, B and C. Module A applies some basic text pre-processing on the abstracts fetched from PubMed followed by entity detections. Syntactic processor is then applied on the pre-processed text. In module B, the mutation mentions in text are recognized and associated with genes and diseases. Finally, in module C, we extract additional information needed for storing the associations in the local databases. These will be described in detail in the next sections.

## Module A

This module includes components for text preprocessing, gene and disease tagging, patient context sentence detection, syntactic processing and matching of patterns.

### Text preprocessing

DiMeX accepts a single or multiple PMIDs as input and processes each abstract individually. We start with extracting the title, abstract text and the MeSH terms from the Medline repository. We use an in-house sentence splitter to split the abstracts into individual sentences. An acronym detector [34] is used to detect possible abbreviated forms to assist the gene and disease tagging steps.

### Gene and disease tagging

We apply a Gene Mention (GM) detector developed in-house. For disease mention detection, we use Pubtator [35], a web-based text mining tool that assists Biocuration by tagging various biological entities. A few enhancements are performed to Pubtator’s results such as a disease is discarded if it is an acronym for which the full form indicates that it is not a disease. For example, in PMID:17591767, “AR” is detected as a disease but the full form “Androgen Receptor” hints that it is a gene. Additionally, “tumor” or “cancer” might be mentioned in general throughout the abstract, without specifying the actual disease every time. These general occurrences, which are annotated by Pubtator as disease terms, are mapped to the closest mentioned disease, which often appears within the same noun phrase.

### Patient context sentence

We identify *Patient Mention* sentences mentioning information about the patients involved in the study, such as total number of participants and demographic information. In general, these sentences also mention the disease central to the study, and so it is important to make the connection with a mutation in those cases in which the disease is not explicitly stated in the sentence that hints to their association. We find that generally the first *Patient Mention* sentence in an abstract is the richest in this type of information. We define a sentence as *Patient Context (PC)* if it is the first *Patient Mention* sentence in the abstract. Example-1 is such a PC sentence:

Example-1:“A total of 453 breast cancer patients and 382 age—and sex-matched controls from Greece and Turkey were analyzed.” (PMID:15330212)

### Syntactic processing

We use BioNex [36] for tokenization of the terms and to perform shallow parsing, which identifies phrases in a sentence. We use the BioNex’s detection of base noun phrases (NPs) and base verb groups (VGs) only. Sometimes, a sequence of base NPs might appear together, connected by conjunctions or prepositions or punctuation marks. We group such sequences of base NPs to form a single longer NP. Similarly, we group the consecutive (with no intervening words or punctuations) base verb groups (VG) together to form a single VG.

In Example-2, the base NPs and base VGs are *NP (The PON1 102V allele)*, *VG (appears)*, *VG (to be associated)*, *NP (an increased risk)*, *NP (prostate cancer)*. Merging of NPs and VGs yields *NP (The PON1 102V allele)*, *VG (appears to be associated)*, *NP (an increased risk for prostate cancer)*.

Example-2: “The PON1 102V allele appears to be associated with an increased risk for prostate cancer.” (PMID:12783936)

As we will discuss in the next subsection on matching of patterns, either a merged NP or its component base NPs can be used to match our extraction patterns. Also, we use the term *Merged NPs* or *Merged VGs* just to emphasize that several base NPs or base VGs have been connected together, respectively.

### Matching of patterns

In this subsection, we will first describe how patterns are specified and then discuss how they are matched against text.

A pattern is specified by a sequence where each component is a noun phrase (NP), a verb group (VG) or a word. An NP mentioned in a pattern sequence can be further instantiated as *NP{head*: *lexical item}*, *NP{contains*: *lexical item}* or *NP<type>*. *NP{head*: *lexical item}* indicates NPs whose head word is the same as the specified lexical item or one of its textual variants. *NP{contains*: *lexical item}* describes NPs that contain the specified lexical item or one of its textual variants. *NP<type>* corresponds to NPs of the specified type (Currently we use only two types: *NP<mutation>* and *NP<disease>*). Similarly, a VG can be instantiated in one of the two ways: *VG_active{head*: *lexical item}* or *VG_passive{head*: *lexical item}*. Thus, in addition to the lexical items, the pattern may require the use of an active or passive VG. In order to match a pattern against text, we consider the BioNex output sequence for a sentence and verify whether this output contains a subsequence that matches the pattern exactly. In this matching, the matched phrases (NP or VG) can either be base or merged phrases. Matching of phrases in patterns that are instantiated with head information (i.e. *NP{head*: *lexical item}*, *VG_active{head*: *lexical item}* or *VG_passive{head*: *lexical item}*) requires the identification of the words that are the heads of the corresponding textual phrases. We take the head of the base NP to be its rightmost word (“allele” in the case of the base NP “The PON1 102V allele”). For a merged NP where the constituent NPs are connected by prepositions, we take its head to be the head of the leftmost base NP (since the others will be modifying the NP to its left). For example, in the case of the merged NP “an increased risk for prostate cancer”, the head is detected to be “risk”. When the constituent NPs are connected by conjunctions, we consider the head word of any of these base NPs. In case of a VG, we take the head word of a base VG to be its rightmost word and the head of a merged VG to be the head of the rightmost base VG. The voice (active or passive) of a verb group is determined based on its rightmost base VG. We determine whether a base VG is active or passive on the basis of the BioNex output. As an illustration, let us consider how the following pattern matches the sentence in Example-2.

$$NP_{1}\;VG\_passive\left\{head:\;associate\right\}\;with\;NP_{2}$$

Here, the merged VG(“appears to be associated”) matches *VG_passive{head*: *associate}* since the head of the rightmost base VG, “associated” is a variant of the lexical item “associate” and the rightmost base VG is passive. NP_1_ is matched with the phrase “The PON1 102V allele” and NP_2_ is matched with “an increased risk for prostate cancer”, which is a merged NP.

For the matching of *NP<type>*, recall that a *NP<type>* can be of two types: *NP<mutation>* or *NP<disease>*. To match the former, the NP must contain a reference to some mutation. This can be either in the form of a specific mutation (e.g., R1699W) or a more generic description, i.e., the head word of one of its constituent NPs must indicate a mutation (e.g., mutation, polymorphism, variant, SNP etc.). To match *NP<disease>* we require the noun phrase to contain a mention of a disease. As an example, consider the following pattern:

NP{head: associate} of  NP<mutation> with NP<disease>

Example-3 refers to a sentence which corresponds to the above pattern. Note that although the entire text is a merged NP, in order to match the pattern, we need to break the merged NP into its constituent NPs. The first base NP matches *NP{head*: *associate}* since its head word is “Association”. The second constituent NP, “the BRCA1 missense variant R1699W”, matches *NP<mutation>* since it contains a specific mutation. The third constituent NP, “a malignant phyllodes tumor of the breast”, matches *NP<disease>* because it includes a mention of a disease. Note that even after we split the longer merged NP into three NPs, *NP<disease>* is still a merged NP.

Example-3: “Association of the BRCA1 missense variant R1699W with a malignant phyllodes tumor of the breast.” (PMID:17574969)

## Module B

Module B consists of components for mutation extraction, mutation to gene association and mutation to disease association. The mutation extraction and mutation-gene association components are fully portable, meaning they can be used in the context of any other problem definition, irrespective of the mutation-disease association.

### Mutation extraction

The mutation extraction employs a list of regular expression patterns to detect mutations containing the three components, i.e., wild-type symbol, mutant-type symbol, and the position. The regular expressions allow for symbols to be single, 3-letter, full mentions of amino acids, or [A,C,G,T] for DNA-bases. Examples of the mutations that are detected using such regular expressions are listed below.

- Protein level mutations: “Ala282Val”, “Asp 327—>Asn”, “T877A”, “Phe153——Ala” etc.
- DNA level mutations: “A3537G”, “4304G> A”, “1066-6T> G”, “-79C/T” etc.

In addition, regular expressions are also used to capture mutations that correspond to insertion, deletion and SNP IDs. Examples of these kinds are listed below.

- Insertions: “5382insC”, “IVS9-5insT” etc.
- Deletions: “9631delC”, “6886delGAAAA”, “IVS19+2delT” etc.
- SNP IDs: “rs1800795”, “ss984046046” etc.

In some cases, conjunctions are part of mutation mentions. For example, in PMID:9466928, “Ala16 >Cys, Thr, Met, Arg, His and Tyr” is mentioned. We detect the conjunctions in this case and generate six mutations: Ala-16-Cys, Ala-16-Thr, Ala-16-Met, Ala-16-Arg, Ala-16-His and Ala-16-Tyr.

We also include extraction of some patterns that are beyond the scope of the above regular expressions. These correspond to mutations that are mentioned in regular text rather than special formats used for mutations as recommended by the Human Genome Variation Society (HGVS) [37]. These extractions are triggered by detection of a pair of amino acids or nucleotides [A,C,G,T]. These are considered as wild-type and mutant-type symbols if an associated mutant position is found. If the mutant position is not mentioned in the same phrase as the wild and mutant-type symbols, then it is usually attached to the phrase with a prepositional phrase (See examples below). We search for specific words, such as *codon*, *position*, *residue* etc. to locate the mutant position. Some examples of a range of mutations extracted using this technique are listed below.

- “A--> C transversion in codon 135”
- “T to C transition at positions 409 and 412”
- “Ser—> Leu change at amino acid 217”
- “termination at codon 3110”
- “guanine-adenine point mutation at nucleotide 2185”

We employ a normalization technique to normalize the mutations into one standard format by matching the wild-type, mutant-type and position. We use “WildType-pos-MutantType” as the standard format for normalization. For example, G5557A and 5557G>A (PMID:22200742) normalize to the same mutation G5557A.

Often, informative sentences refer to a mutation-disease association in terms of alleles or genotypes rather than mutations. For this reason, we detect these mentions and match them to the corresponding mutation that might be mentioned elsewhere in the abstract. In Example-4, the association with gastric cancer is referred using the allele 194Trp, whose corresponding mutation is Arg194Trp found in the abstract.

Example-4: “XRCC1 194Trp allele significantly increased the risk of gastric cancer and also associated with risk of gastric cardia carcinoma and promoted distant metastasis of gastric cancer.” (PMID:20863780)

Allele mentions are usually similar to mutation mentions except sometimes they may not specify both the wild-type and mutant-type. For instance, in Example-4, *194Trp allele* is mentioned without a wild-type associated with it. In this case, we match the allele to the mutation *Arg194Trp* which is mentioned elsewhere in the abstract. However, it is more common to find both wild-type and mutant-type together in a genotype mention. Example-5 demonstrates one such case where *AG* genotype represents the wild-type and mutant-type pair. We will still need to associate it with the actual mutation. To identify the corresponding mutation in the abstract, we look for the mutations whose wild-type and mutant-type match with the genotype nucleotides. If multiple of such mutations are found, we associate the closest one with the genotype. For example, the *AG* genotype is matched with the mutation *+49G/A* from an earlier sentence in the same abstract. The other genotypes in the same sentence (*GG* and *AA*) are also matched with *+49G/A* because they are mentioned along with *AG* and share the same nucleotide symbols.

Example-5: “In HCC and CHB groups, the genotype frequency was 40.3% and 50.0% for GG, and 59.7% and 50.0% for AG+AA, respectively, while the genotype frequency was 61.8% for GG and 38.2% for AG+AA in the control group." (PMID:20813679)

### Mutation to gene association

Once the mutations are detected, we associate them with their relevant genes. In many cases, this is straightforward as the mutations and the corresponding genes are mentioned close to each other. For example, when both the mutation and the gene appear in the same *merged NP*, we associate them with high confidence. Example-6 presents one such case where “C-2123G”, “G-1969A”, and “T715P” are associated with SELP, and “Met62Ile” is associated with PSGL-1. Please note that we do not detect the generic references to genetic variations as mutation mentions, such as the phrase “VNTR variants” in this case.

Example-6: “Our aim was to evaluate the contribution to CHD of the following SNPs: C-2123G, G-1969A and T715P in SELP, Met62Ile and the VNTR variants in PSGL-1 gene in a North African population from Tunisia.” (PMID:20376705)

Even in situations when a particular mutation occurrence does not have an accompanying gene in the same sentence, we have noticed that, often, the gene is mentioned in the same NP or same *merged NP* with the mutation at least once in some other sentence in the abstract. We propagate the gene detected in these latter cases to all occurrences of the mutation in the rest of the abstract.

There are cases, however, of mutations that do not appear together with their corresponding genes in any sentence of the abstract. If a gene is mentioned anywhere in the whole abstract and it is the only gene mentioned, then we associate it with the mutation. However, if multiple genes are mentioned in the abstract, we look for ones that occur together with a mutation-specific term, such as “variant”, “mutant”, “variation”, “mutation”, “polymorphism”, “alteration” or “SNP” in the same *merged NP*. We call this occurrence of the gene and the mutation specific term a *gene-mutation pairing*. For any detected mutation, we associate it to the gene mentioned in the closest *gene-mutation pairing* that occurred previously in text, either in the same sentence or any sentence before. Once a mutation has been associated with a gene, the association is propagated to every occurrence of the mutation in that abstract. In Example-7a, the gene ELAC2/HPC2 is detected as having a *gene-mutation pairing* because of the phrase “mutations of the ELAC2/HPC2 gene”. The immediately following sentence, which is shown in Example-7b, has a mutation Glu622Val that does no co-appear with a gene. Applying our rule, Glu622Val is associated with ELAC2/HPC2.

Example-7a: “Here, we screened for mutations of the ELAC2/HPC2 gene in 66 Finnish HPC families.” (PMID:11507049)

Example-7b: “Several sequence variants, including a new exonic variant (Glu622Val) were found, but none of the mutations were truncating.” (PMID:11507049)

Finally, for a detected allele, we first identify the corresponding mutation and then the gene associated with the mutation. We then associate the allele with the gene.

### Mutation to disease association

Once the mutations are extracted and paired with genes, the next step is to find the association of mutations with diseases to complete the extraction of the mutation, gene and disease triplet.

An association between a mutation and a disease can be conveyed in different ways in a sentence, either explicitly or implicitly. Based on our preliminary studies, we have observed that there are six types of sentence structures that are commonly used to specify such associations. For each of these cases, we first describe the type of sentence structure and the patterns used to identify them. Next, we describe how the mutations and the diseases, which will be associated, are identified. Since the technique of extraction for the mutation and the disease may be specific to the sentence structure types, we will discuss them after describing each sentence structure type.

#### (i)Association sentence type

There are several lexico-syntactic structures that are used to denote an association between two entities. We call these structures as *Association Sentence Type*. Based on our preliminary study, we identified a few common ways that are employed to describe associations between a mutation and a disease and capture them by defining a set of lexico-syntactic patterns. Matching a sentence against these patterns allows us to identify sentences that are Association Sentence Type. We associate each pattern with a trigger word, where the trigger word appears in the lexical instantiation within the pattern. For example, the following four patterns are defined for the lexical trigger “associate”.

$$NP_{1}\;VG\_passive\left\{head:\;associate\right\}\;with\;NP_{2}$$

$$NP_{1}\;VG\_active\left\{head:\;associate\right\}\;with\;NP_{2}$$

$$NP\left\{head:\;associate\right\}\;of\;\;NP_{1}\;with/and\;NP_{2}$$

$$NP\left\{head:\;associate\right\}\;between\;\;NP_{1}\;and\;NP_{2}$$

As noted earlier, Example-2 matches the first of these rules, where NP_1_ matches the base NP “the PON1 102V allele” which will allow us to identify the mutation and NP_2_ matches the merged phrase “an increased risk for prostate cancer” which is used to identify the disease.

In addition to “associate”, we use several other triggers words “contribute”, “correlate”, “relationship”, and “effect” and their associated patterns. Many of these words do not necessarily indicate associations but could indicate closely related concepts such as causality. Also, many of these words impose strong subcategorization requirements on prepositions that appear in their arguments. Instead of using multi-word triggers, the prepositions are mentioned in the patterns themselves. For example, one such pattern is *NP{head*: *effect} of NP*_*1*_ *in/on NP*_*2*_ which is matched by Example-8.

Example-8: “Synergistic effect of stromelysin-1 (matrix metalloproteinase-3) promoter (-1171 5A-> 6A) polymorphism in oral submucous fibrosis and head and neck lesions.” (PMID:20630073)

The full list of patterns with their trigger words used in this study is available in S1 File. Once it is determined that a sentence corresponds to Association Sentence Type, DiMeX extracts the mutation and the disease and makes an association between them.

Mutation detection: We look for mutation mentions in either of NP arguments in these patterns, namely in *NP*_*1*_ or *NP*_*2*_. To do so, we check whether either of the NPs corresponds to *NP<mutation>*. Note that a phrase matching *NP<mutation>* need not contain a specific mutation. In this case if the phrase contains a gene name, we extract the referent mutation from the closest previous sentence where that mutation is already associated with that gene. For example, *NP<mutation>* phrase, “PON1 mutations”, in Example-9 matches NP_1_ of the third pattern and the procedure described above allows us to extract the referent, I102V, from a previous sentence in the abstract.

Example-9: “Multivariable analysis was used to investigate the association of known and new PON1 mutations with incident prostate cancer in 1569 cancer-free men in the cohort followed for 9–14 years.” (PMID:12783936)

Disease detection: Like in the case of mutations, we look for a disease in the *NP*_*1*_ or *NP*_*2*_ arguments of the patterns. Thus, we look for a phrase matching *NP<disease>*. Once the disease is extracted, the system reports a mutation-disease pair for each mutation. In Example-9, the *NP<disease>* phrase matches NP_2_ of the pattern and allows us to extract the pair <I102V, prostate cancer>.

#### (ii) Comparison sentence type

Association between mutations and diseases can sometimes be inferred from sentences that describe experimental results comparing two cases (e.g., mutated and wild-type forms) with some observed value. In Example-10 below, the comparison is between the wild-type and the mutated form of NDPK-A and in Example-11, the comparison is drawn between breast cancer patients and healthy volunteers.

Example-10: “Compared with its wild-type, NDPK-A (S120G) appears more effective in promoting neuroblastoma metastasis.” (PMID:15280446)

Example-11: “Statistical analysis of cases with HFE H63D phenotype showed significant difference between breast cancer and healthy volunteers (P = 0.02).” (PMID:16503999)

As these examples show, there are different syntactic constructs that can be used for the comparison sentence type. Additionally, a comparison can be indicated by different triggers words such as “compare” (Example-10) or even “difference” (Example-11). The above two sentences match the following two patterns.

$$VG\_active\left\{head:\;compare\right\}\;to/with\;\;NP_{1}\;,\;\;NP_{2}\;VG\_active\;NP\left\{contains:\;more/less\right\}\;in\;NP_{3}$$

$$NP_{1}\;VG\_active\;NP\left\{head:\;difference\right\}\;between\;\;NP_{2}\;and\;NP_{3}$$

Other patterns with their associated triggers can be found in S1 File.

Mutation detection: The mutation can be found in either of the NPs that are used in comparison (as in Example-10) or in the NP concerned with the observation (Example-11). Thus, if any of these NPs matches *NP<mutation>*, we extract the mutation from it using the procedure we described in case of Association Sentence Type.

Disease detection: Since the disease can also be found in any of the NPs, we look to see if any of the three NPs matches *NP<disease>*. Thus, in Example-10 we obtain “neuroblastoma” from the “observation/observed value” NP and extract breast cancer in Example-11 from one of the “comparison” NPs, NP_2_.

#### (iii) Statistical sentence type

Sentences that mention statistically significant results suggest some sort of association. Mentions of P or OR (odds ratio) value indicate the reporting of statistically significant results. Thus we define any sentence that reports these values to be of Statistical Sentence Type. When such a sentence mentions a mutation and a disease, we assume that the experimental results indicate an association between the two. Example-12 is a sentence of this kind.

Example-12: “For the -37C—> A polymorphism, the median PFS was 30.7 weeks in the C(-) 37A group, 24.7 weeks in the A(-) 37A group, and 23.3 weeks in the C(-) 37C group (P = 0.043).” (PMID:20226083)

In many cases, sentences mentioning P-value also fit the pattern for the previously mentioned association and comparison sentence types. Thus, if a sentence is not detected as either association or comparison sentence type, but if it contains a P or an OR value, it is marked as a statistical sentence.

Mutation detection: Based on our observations of these type of sentences, we have seen that the mutation is always mentioned in these statistical sentences (e.g., as is the case in Example-12). Thus, we search for a noun phrase that matches *NP<mutation>* in the detected statistical sentence, and extract the mutation as before.

Disease detection: We only extract a disease if a mutation was found in the detected statistical sentence. If there is no mention of a disease in the sentence (i.e. there is no *NP<disease>*), we assume that the disease is implicit in context. Since we take the disease mentioned in the *Patient Context (PC*) sentence (introduced in Module A) to be the central disease of the study, we assume the experimental study reported in the paper must be associating the mutation with this particular disease. Thus, we look for it in a PC sentence, provided such a sentence occurs before the current sentence. If the disease is not found in a PC sentence, we look for the central disease at other rhetorical zones in the abstract in the following order: title, conclusion sentence(s) and introduction sentences (determination of whether a sentence is in Introduction or Conclusion is described in “Extraction and rhetorical zones” subsection of Module-C). For instance, Example-12 does not mention a disease, and so we extract the disease “NSCLC” from the title *“RRM1 single nucleotide polymorphism -37C—>A correlates with progression-free survival in NSCLC patients after gemcitabine-based chemotherapy”*. Thus, Example-12 yields the pair <-37C—> A, NSCLC>.

#### (iv) Mutation Found sentence type

Sometimes, a study may not have enough participants to produce results that are statistically significant, though the results may have clinical significance. In the abstracts of such articles, usually there are sentences that mention mutations that were “found/detected” for a specific group of patients. While the authors may not draw firm conclusions, we are still interested in extraction from these cases as well, since we believe the authors are interested in the connection between the mutation and the disease. Example-13 shows one such sentence.

Example-13: “We have investigated German breast- and/or ovarian-cancer families and detected a recurrent carboxy-terminal BRCA1 mutation, 5622C > T, using PCR-based restriction assay and haplotype analysis.” (PMID:11260866)

To detect Mutation Found sentences, we use several trigger words such as “detect”, “identify”, “analyze”, and “screen” and associated patterns. These patterns essentially indicate that the object (or theme) of the trigger words is *NP<mutation>*. Thus, only the fragment of the sentence, which indicates that a mutational phrase is the theme of detection, will be matched with the patterns. A few sample patterns whose trigger word is “detect” are listed below. A complete listing is found in S1 File.

NP{head: detect} of  NP<mutation>

NP<mutation> VG_passive{head: detect}

VG_active{head: detect} NP<mutation>

Mutation detection: We usually extract the mutation from the noun phrase that matches the *NP<mutation>* of the pattern. But sometimes, as in Example-14a, the Mutation Found sentence might only mention a number of mutations and invariably then the actual mutations are listed immediately after. We look for the mutations in the following one or two sentences. Example 14b is the sentence that follows Example-14a and mentions the actual mutations.

Example-14a: “Three different mutations, resulting in truncation of the BRCA2 protein, were detected in 3 different families.” (PMID:9133456)

Example-14b: “They were 9474insA (exon 24, termination at codon 3110), C8729A (exon 20, S2834 ter) and 982del4 (exon 9, termination at codon 275).” (PMID:9133456)

Disease detection: We first attempt to extract the disease from the Mutation Found sentence. However, if there is no mention of a disease in the sentence, we look for it in other places in the following order: Patient Context (PC) sentence, title, conclusion sentences and introduction sentences.

Example-14c shows the PC sentence from which the disease “breast cancer” is extracted and linked to the mutations of Example-14a and Example-14b to yield the pairs: <9474insA, breast cancer>, <C8729A, breast cancer>, and <982del4, breast cancer>.

Example-14c: “Germline mutations of BRCA2 were examined in 20 Japanese breast cancer families without BRCA1 mutations, including one demonstrating cancer development in a male.” (PMID:9133456)

#### (v) Title sentences

The title of an article typically summarizes the central topic of discussion. Therefore, if the title contains mutation and disease mentions, we infer that the article is about an association between the two. In particular, we assume the study reported in the paper is making an association between a mutation and a disease mentioned in the title even if it does not follow our patterns of association. For example, the following title in Example-15 yields a possible association of < IVS1 -27G> A, prostate cancer >.

Example-15: “KLF6 IVS1 -27G> A variant and the risk of prostate cancer in Finland.” (PMID:17125911)

#### (vi) Conclusion sentences

Similar to the title, a conclusion sentence usually summarizes the results or the key points of the article. Therefore, if mutations and diseases are found in conclusion sentences, we again assume an association between them. Strategies for classifying a sentence as a conclusion sentence is detailed in the “Extraction and rhetorical zones” subsection under Module C of this article.

## Module C

Our intention is to make all the extracted information available by storing them in a database, so that they can be easily searched and manipulated. This section describes the details of the database and the additional information that we include in the database.

### Database creation

DiMeX extracts triplets of mutations, genes and diseases (<M,G,D>) from various abstract sentences along with additional useful information that can be used to search and display results more efficiently. Since the information is extracted from the literature, the appropriate document can also be linked directly to the database entities for display of the context around the extracted information. A database is generally more amenable to electronic processing. For these reasons, we have created a database to house the extracted information that is able to support different use case scenarios, such as:

1. Given a gene, search for possible mutations of the gene that are associated with different diseases.
2. Given a mutation, find all possible diseases it is associated with.
3. Given a disease, find possible <gene, mutation> pairs that might be affecting the disease.
4. Given a <gene, mutation> pair, find all associated diseases from articles that have conducted experimental studies on the given mutation.
5. Given a <mutation, disease> pair, find the description of the population (e.g., number of patients/controls, location of population, etc.) involved in relevant studies.
6. Given a <mutation, disease> pair, find how the mutation affects the disease, such as overall outcome or drug sensitivity.
7. Given a <gene, mutation> pair, find all associated diseases from meta-analysis or review articles.

Scenarios (*i)* to (*iii)* require simple queries of the database. Scenarios (*iv)* to (*vii)* are a little more complex, in that they involve the additional information extracted from the literature for the <M,G,D> triplets, such as the rhetorical zone of the extracted triplet (*iv*), the patient and population related information (*v*), the manner in which the mutation affects the associated disease (*vi*), the study type such as meta-analysis or review (*vii*,) etc. Please note that in these cases, instead of a pair, we can also search for a single entity (e.g., mutation or gene or disease) to obtain the desired result set. In all the above scenarios, the results can be further filtered and sorted according to the need of the user. The extraction of the additional information is described in next sections.

#### Extraction and rhetorical zones

A triplet may be extracted from any rhetorical zones or sections within the abstract text. Information about the rhetorical type of a sentence might be important because, for example, a triplet extracted from the results or conclusion sections of the abstract might be of greater interest since the triplet is likely to be central to the study or experiment being done in the article, and potentially a novel information. In contrast, a triplet coming from the background section often refers to some previous study and thus does not provide novel information. Our approach categorizes each sentence of each abstract as one of five rhetorical types: Title, Introduction/Background, Methods/Aims, Results, and Conclusion. If the abstract is already sectioned into these rhetorical zones, we detect and use this information to assign the sentences to the corresponding zones. Otherwise, we identify the zone boundaries in the abstract. Users of DiMeX can use the rhetoric zone information to prioritize the extractions they want to see first. Recall that identification of the zones in the abstract is also used during the mutation to disease association, as already described.

In the database, we associate a rhetorical zone with each extracted triplet. If the same triplet is extracted from multiple zones, we apply a simple ranking of the zones and set the triplet’s extraction zone to the top rank. The ranking follows this order: Results, Conclusion, Title, Method, and Introduction. For example, if a triplet is extracted from both the Introduction and the Results zones of the abstract, the database indicates that the triplet is extracted from Results.

There are previous works [38–43] that use different approaches to classify sentences into rhetorical zones or sections. These tools classify each sentence into one of the rhetorical zones using machine learning based methods which require sufficient training data. As we could not download any of these tools, we have developed our own module to perform the task. Our method differs from the above mentioned tools in two ways. Firstly, it is based on some simple heuristic rules. Secondly, instead of treating each sentence separately, we look at the abstract as a whole and set the boundaries of the sections. In other words, we check whether a sentence marks the boundary of a section or not. The position of the sentences and certain keywords are used for this purpose. The start of the Introduction section and the end of the Conclusion section are obvious as the beginning and the end of the abstract itself. For any other pair of consecutive sections, it is sufficient to find the start of the section that follows. To detect the end of the Introduction section and the start of the Method section, we look for phrases such as “we have analyzed”, “we studied”, “our aim is to”, etc. If no such sentence is found, we assume that the Method section starts after three Introduction sentences. Similarly, phrases such as “we found that”, “the results indicate that”, “our findings exhibit that”, “we have shown that”, etc. are strong indicators of a shift from the Method section to the Results section. Finally, to mark the ending of the Result section and the start of the Conclusion section, we look for phrases like “In conclusion”, “We conclude by”, etc. In case we fail to find such cases, we assume that the very last sentence of the abstract forms the Conclusion section. Please note that there could be more than one sentence for each of the sections. Table 1 shows one example sentence for each of the rhetorical zones identified by our system.

**Table 1:** Example sentences for different sections from PMID:10810408.

Rhetorical zone/section	Example sentence
Title	Missense alterations of BRCA1 gene detected in diverse cancer patients.
Introduction/Background	BRCA1 gene mutations may also be related with other types of cancers such as prostate cancer and colorectal cancer.
Methods/Aims	We used PCR-NIRCA and PCR-SSCP methods for screening the BRCA1 mutation hot regions, exons 2, 5, 11, 16 and 20.
Results	We have identified a rare sequence variant, A3537G (Ser 1140Gly) in a B cell lymphoma patient and two polymorphisms, A1186G (Gln356Arg) in a brain cancer patient and A3667G (Lys1183Arg) in a germline tumor patient.
Conclusion	In conclusion, 3 missense alterations of BRCA1 gene have been identified in cancers other than breast cancer.

#### Patient related information

For mutation-disease associations, it is helpful to know information related to the patients, such as the size of the experimental patient population and the control population, the race or nationality etc. This additional information is extracted from literature and associated with the abstract. Patient-related information is commonly present in *Patient Context* sentences (introduced in Module A). Consider the *Patient Context* sentence that we have already presented in Example-1:

“A total of 453 breast cancer patients and 382 age- and sex-matched controls from Greece and Turkey were analyzed.” (PMID:15330212)

We extract the following information from the above sentence:

- Patients: 453, Controls: 382
- Population: Greece and Turkey

We extract the region of the population or nationality using a pre-compiled list of country names, their adjectival forms and demonyms (names given to residents of a place, e.g., Sri Lankan, Chinese, Peruvian etc.). To detect the patient cohort size, we use predefined patterns.

#### Disease related outcome

In addition to extracting diseases, our system can detect several types of outcomes and relate them to the extracted mutations. For example, it detects phrases like *disease-free survival (DFS)*, *progression-free survival (PFS)*, *overall survival*, as well as phrases that denote *risk* or *metastases* of a specific disease. This information provides insight into how the mutations affect the diseases. For instance, in Example-16, the SNP *rs1878022* is found to be associated with *poor overall survival* (outcome) of *non-small cell lung cancer* (disease).

Example-16: “SNP rs1878022 in the chemokine-like receptor 1 (CMKLR1) was statistically significantly associated with poor overall survival in the MD Anderson discovery population.” (PMID:21483023)

DiMeX also detects phrases that denote the impact of a drug in treatment or therapy, such as *resistance* or *sensitivity* to a drug. For instance, in Example-17, DiMeX will report that the mutation *538G>A* of gene ABCC11 is related to *MTA sensitivity*. This type of information is helpful in understanding the efficacy of drugs in the treatment of diseases.

Example-17: “The A/A group showed a significant reduction in the IC (50) of MTA compared with the combined G/G and G/A groups, indicating that the SNP (538G> A) in the ABCC11 gene is an important determinant of MTA sensitivity.” (PMID:20718756)

Extraction of outcome information or the impact of drugs in cancer treatment is currently limited by the use of trigger words, and we are focusing on improving the detection. The list of patterns used to detect disease related outcomes is available in S2 File, along with the list of phrases detected from the DiMeX database.

#### Meta-analysis and review

Our system also detects whether the conducted study in the article is a meta-analysis. To detect meta-analysis studies, we search for textual variations of the keyword “meta-analysis” (e.g., meta analysis, meta-analyses, meta-analyzing, etc.), as well as for number of publications or studies the authors has reviewed. For instance, Example-18 is a title which mentions that the work is a meta-analysis. A later sentence in the same abstract mentions *“A total of 11 publications containing 12 studies including 10,137 cases and 15,566 controls were identified”*. Combining the extracted information from these two sentences, we conclude that this abstract talks about a meta-analysis study with data from 11 publications and 12 studies, involving 25,703 subjects, information which is stored in the database for this abstract.

Example-18: “Lack of an association between a functional polymorphism in the interleukin-6 gene promoter and breast cancer risk: a meta-analysis involving 25,703 subjects.” (PMID:20043205)

Similar to detecting meta-analysis studies, we detect whether the article is a review study. We look at the MeSH (Medical Subject Headings) terms of the Medline citation to see if it contains the keyword “Review” under the tag PT (Publication Type).

We extracted <M,G,D> triplets and the above described additional information from a subset of abstracts that were retrieved for a PubMed query “*cancer[tiab]) AND (mutation[tiab] OR variant[tiab] OR polymorphism[tiab])*”. We built a database with all this information and the results can be downloaded as well as queried from the web interface.

## Evaluation

Our system involves 3 separate modules that detect mutations (M), associate mutations with genes (<M,G>) and with diseases, leading to the extraction of <M,G,D> triplets. Mentions of mutation-disease association refers to <M,G,D> extraction throughout the rest of the article. Although the main focus of this work is to associate mutations with diseases, we have evaluated our system on all three tasks. Any mistakes made in the mutation-gene association or mutation detection will impact the mutation-disease association, as the accuracy of the later requires all three pieces to be correctly extracted. We have used several datasets for evaluation, which were externally developed. Whenever other tools were evaluated using any of these datasets, we compared our results with them.

DiMeX’s performance in extracting mutations and associating them with genes and diseases has been evaluated using annotated gold dataset from two different sources. The first dataset is a manually annotated corpus from the BioMuta [6] project, which we will call *BiomutaC*. BioMuta is an integrated database aiming to provide a framework for automated and manual curation and integration of cancer-related variations. This set is not specific to any particular cancer. Although BioMuta considers full text, the annotation included in this data set is based on abstract text alone. *BiomutaC* contains 62 abstracts with 119 mutation-gene-disease association triplets.

A second collection of two publicly available datasets described in Doughty et al. [16] was used for evaluating DiMeX. These allowed for the comparison of our work with previously published results. We will call the two sets *PCa_filtered_UD* and *BCa_filtered_UD*, corresponding to abstracts from prostate cancer (PC) and breast cancer (BC), respectively. There are 97 and 132 abstracts in *PCa_filtered_UD* and *BCa_filtered_UD*, respectively. Originally, we wanted to evaluate DiMeX on the exact datasets *PCa_filtered* (113 abstracts) and *BCa_filtered* (147 abstracts) that were used in [16], giving us a chance to directly compare performances with already published results. However, instead of the exact datasets, we received two larger datasets from the authors and the filtering criteria to regenerate the datasets they have used. The first author of [16] clarified the filtering criteria to remove either an abstract or a mutation based on certain conditions. Abstracts were removed if:

- Curator and validator disagreed.
- Wild-type amino acid (wtaa) and mutant-type amino acid (mtaa) were the same.
- Contained incomplete mutation (missing wtaa, mtaa, mutant position, or gene).
- Mutant position started with a “-”.
- wtaa or mtaa was “INS”, “DEL”, “IVS”, “DUP”, “INDEL”.

Similarly, mutations were removed if:

- Wild-type or mutant-type were stop codons (XAA, X, Stop)
- Detected mutation was not related to target disease (PC or BC).

Although we have strictly applied the filtering criteria on the larger datasets, we ended up with a slightly different set of abstracts than the ones referred to in [16]. That’s why we had to rename them differently (UD stands for University of Delaware). We have applied both DiMeX and EMU on these two datasets (*PCa_filtered_UD* and *BCa_filtered_UD*) so that the evaluation results are directly comparable.

To evaluate mutation-gene association, we used the same sets that we used for mutation-disease association, namely *BiomutaC*, *PCa_filtered_UD*, and B*Ca_filtered_UD*. We evaluated DiMeX’s mutation detection using three different corpora. The first one is Mutation Finder [20], which we will refer to as *MF*. We chose *MF* for this evaluation as it is the most popular benchmark which has been used in Jimeno Yepes et al. [44] to compare different mutation detection systems. The *MF* set consists of 910 point mutation mentions from 508 abstracts. One thing to note is that MF identifies only point mutations, whereas our system extracts deletion, insertion, frameshift and dbSNP identifiers as well. For comparison purposes, we only considered the point mutations. To test the wide coverage of mutation extraction, we evaluated our system on two other corpora: tmvar [28] and Variome [33]. The tmVar corpus contains 464 mutation annotations from 166 abstracts. The Variome is a corpus of 10 full text publications which includes both the specific mutation mentions as well as generic references to mutations such as “mutations” or “somatic mutations”. As we detect specific mentions only, we identified 118 instances of specific mutation mentions in the annotated corpus and used for evaluation. There are other corpora available too, such as OSIRIS [25] and Thomas [45], which could be used for evaluating mutation extraction and mutation normalization to dbSNP entries.

Table 2 summarizes the six datasets used for evaluation in this study. *BiomutaC* is available as supplementary information (S1 Dataset). As indicated above, both *PCa_filtered_UD* and *BCa_filtered_UD* were created by applying appropriate filtering criteria on two larger datasets. These datasets were obtained by contacting the corresponding author of Doughty et al. [16]. The list of PMIDs of *PCa_filtered_UD* and *BCa_filtered_UD* are included as supplementary information (S2 Dataset). Together with the above mentioned filtering criteria and the original annotated datasets, which can be obtained by contacting the authors of [16], our evaluation data can be recreated in their entirety. The MF dataset is available at http://mutationfinder.sourceforge.net/. The Variome corpus is available at http://www.opennicta.com.au/home/health/variome. The tmVar corpus is available at http://www.ncbi.nlm.nih.gov/CBBresearch/Lu/Demo/tmTools/#tmVar.

**Table 2:** Summary information of the datasets used for evaluation purposes.

Name of dataset	Used for tasks of	Used for evaluation of	Size
BiomutaC	<M,G,D> & <M,G>	DiMeX	62 abstracts (119 <M,G,D> triplets)
PCa_filtered_UD	<M,G,D> & <M,G>	DiMeX & EMU	97 abstracts (170 <M,G,D> triplets)
BCa_filtered_UD	<M,G,D> & <M,G>	DiMeX & EMU	132 abstracts (216 <M,G,D> triplets)
MF	M	DiMeX	508 abstracts (910 point mutations)
Variome	M	DiMeX	10 full text articles (118 mutations)
tmVar	M	DiMeX	166 abstracts (464 mutations)

<M,G,D> refers to mutation-disease associations. <M,G> refers to mutation-gene associations. M refers to mutation detection.

### Evaluation metrics

We counted true positives (TP), false positives (FP), and false negatives (FN), and used the standard information retrieval metrics of Precision (P), Recall (R), and F-measure (F) for performance evaluation, where P = TP/(TP+FP), R = TP/(TP+FN) and F = 2PR/(P+R).

## Results and Discussion

### Evaluation using the *BiomutaC* set

DiMeX’s performance on the *BiomutaC* set is summarized in Table 3. We have separately calculated the precision, recall and F-measure for mutation-gene (<M,G>) and mutation-disease associations, which eventually refers to the whole triplet <M,G,D> extractions. We achieved high precision and recall on this set, with an F-measure of 0.88 for <M,G,D> and 0.93 for <M,G>. In-depth analysis of the false positive (FP) and false negative (FN) cases revealed that most of the errors are due to gene mention and disease detection problems. For example, in five cases, the gene mention detector failed to detect the target gene name. Because the correct gene was missed, our algorithm linked the wrong gene, contributing towards both FP and FN. In a handful of cases, the mistakes were made in the mutation detection, mutation-gene association or mutation-disease association. Overall, only two mutations were not detected by DiMeX that contributed towards FN. In only one case, our algorithm associated a wrong gene with a mutation. The main focus of our work, mutation-disease associations, failed only in two cases.

**Table 3:** DiMeX’s performance of mutation-disease (<M,G,D>) and mutation-gene (<M,G>) association on *BiomutaC* set.

Dataset	DiMeX performance in <M,G,D> extraction			DiMeX performance in <M,G> extraction		
	P	R	F	P	R	F
BiomutaC	0.87	0.89	0.88	0.90	0.95	0.93

### Evaluation using the PC and BC related sets

Table 4 lists the evaluation results for mutation-disease (<M,G,D>) associations using the *PCa_filtered_UD* and *BCa_filtered_UD* sets. Since, these sets of abstracts were only annotated for prostate cancers (PC) and breast cancers (BC), the extracted triplets with any disease other than PC or BC were not considered in our evaluation. F-measures of 0.91 and 0.89 were achieved by DiMeX for the *PCa_filtered_UD* and *BCa_filtered_UD* set, respectively with the PCa_filtered_UD set yielding higher precision and recall. EMU [16] yielded F-measures of 0.75 and 0.67 for the *PCa_filtered_UD* and *BCa_filtered_UD* sets, respectively. We performed paired t-test to check for statistical significance on these datasets (p = 0.002 and p = 0.00007, respectively). We have also evaluated DiMeX for <M,G> extraction on the same sets (see Table 5). F-measure was 0.94 for both *PCa_filtered_UD* and *BCa_filtered_UD* sets. EMU scored F-measure of 0.76 and 0.68 on the *PCa_filtered_UD* and *BCa_filtered_UD* sets, respectively. Again statistical significance was achieved with p = 0.003 and p = 0.0007, respectively.

**Table 4:** DiMeX’s performance of mutation-disease association (<M,G,D>) on PC and BC related sets and comparison with EMU’s performance.

Datasets	DiMeX performance in <M,G,D> extraction			EMU performance in <M,G,D> extraction		
	P	R	F	P	R	F
PCa_filtered_UD	0.95	0.88	0.91	0.76	0.75	0.75
BCa_filtered_UD	0.93	0.85	0.89	0.64	0.71	0.67

**Table 5:** DiMeX’s performance of mutation-gene association (<M,G>) on PC and BC related sets and comparison with EMU’s performance.

Datasets	DiMeX performance in <M,G> extraction			EMU performance in<M,G> extraction		
	P	R	F	P	R	F
PCa_filtered_UD	0.96	0.92	0.94	0.77	0.75	0.76
BCa_filtered_UD	0.94	0.94	0.94	0.65	0.72	0.68

Since *BCa_filtered_UD* showed a little lower precision (0.93), recall (0.85) and F-measure (0.89) than *PCa_filtered_UD* in <M,G,D> extraction, we analyzed the errors on this set. Similar to the *BiomutaC* set, analyzing the *BCa_filtered_UD* results revealed that most FPs and FNs are due to mistakes in gene or disease detection. There are six cases of mutations being erroneously detected, mostly described in regular text rather than standard formats used for mutations. In four cases, the mutation detection component missed the mutations entirely, contributing to FN. For example, in PMID:10207667, the phrase *“This germ line mutation leads to the replacement of isoleucine by asparagine”* gives the wild-type and mutant-type but the codon position is mentioned in the previous sentence. There were five cases of a wrong gene associated with a mutation. Similarly, there were several cases of mutation-disease associations being erroneously inferred, mostly because multiple diseases were mentioned in the context of the abstract and our extraction technique attached the wrong disease to the mutations. DiMeX failed to extract a handful of mutation-disease associations, which contributed towards lower recall as well. Many of these associations were described using patterns that were not part of our pre-compiled list of sentence patterns. In some cases, our definition of an association might have differed from the annotation guidelines used for *BCa_filtered_UD*. For instance, Example-19 sentence sets the context of the abstract with mentions of what was studied. But later in the abstract, there were no definite conclusions about the association of the mutation Val384Asp with breast cancer. Our system did not extract this association, but it was included in the annotated gold set.

Example-19: “A case-control study was taken to investigate the role of Val384Asp in hMLH1 gene in developing these four carcinomas. 233 colorectal, 273 gastric, 90 esophageal and 111 breast cancer patients were included, as well as 268 healthy individual served as controls.” (PMID:15769334)

The *PCa_filtered_UD* dataset also showed similar distribution of errors for the false positives and false negatives.

### Evaluating mutation detection

We have evaluated the performance of DiMeX’s mutation detection component on three corpora: MF, Variome and tmVar. Table 6 provides DiMeX results on these corpora along with the results of the tools MF [20], EMU [16], tmVar [28], and SETH [30]. The results in Table 6 for the other tools are published results from the original papers, the SETH tool website (https://rockt.github.io/SETH/) as well as from [44]. The latter reports a comprehensive study of the mutation detection tools on the Variome corpus which covers a variety of mutation types. We ran tmVar, SETH and EMU on the Variome corpus and found that DiMeX’s performance was not statistically significantly different from them. In order to include EMU’s performance on MF, we had to consider performance for normalized mutations, since EMU only reports results for normalized mutations, where multiple occurrences of the same mutation are normalized to one entry and evaluation is done on the normalized mutation instead of considering all occurrences.

**Table 6:** Evaluation of mutation detection systems on various datasets.

Tool	Performance measures	Corpus		
		MF (MF mutations normalized)	Variome	tmVar
MF	P	0.98 (0.98)	0.94	-
	R	0.82 (0.81)	0.16	-
	F	0.89 (0.89)	0.24	-
EMU	P	- (0.99)	0.97	-
	R	- (0.81)	0.76	-
	F	- (0.89)	0.85	-
tmVar	P	0.99 (0.98)	0.97	0.91
	R	0.90 (0.84)	0.91	0.91
	F	0.94 (0.90)	0.94	0.91
SETH	P	0.98 (0.97)	0.99	0.94
	R	0.82 (0.81)	0.76	0.81
	F	0.89 (0.88)	0.86	0.87
DiMeX	P	0.99 (0.98)	0.96	0.94
	R	0.89 (0.89)	0.92	0.89
	F	0.94 (0.93)	0.94	0.91

The values in precision (P), recall (R) and F-measure (F) for tools other than DiMeX are obtained from comparisons performed in [44] and published results in [20], [28] and SETH tool website. A dash (‘-’) indicates unavailability of data. The tools are MutationFinder (MF), Extractor of Mutations (EMU), tmVar and SNP Extraction Tool for Human Variations (SETH) and DiMeX. For the MF corpus, the results in parenthesis represent evaluation on normalized mutations where multiple occurrences of the same mutation are normalized to one entry.

### Creation of the text-mined database

**Fig 2:**
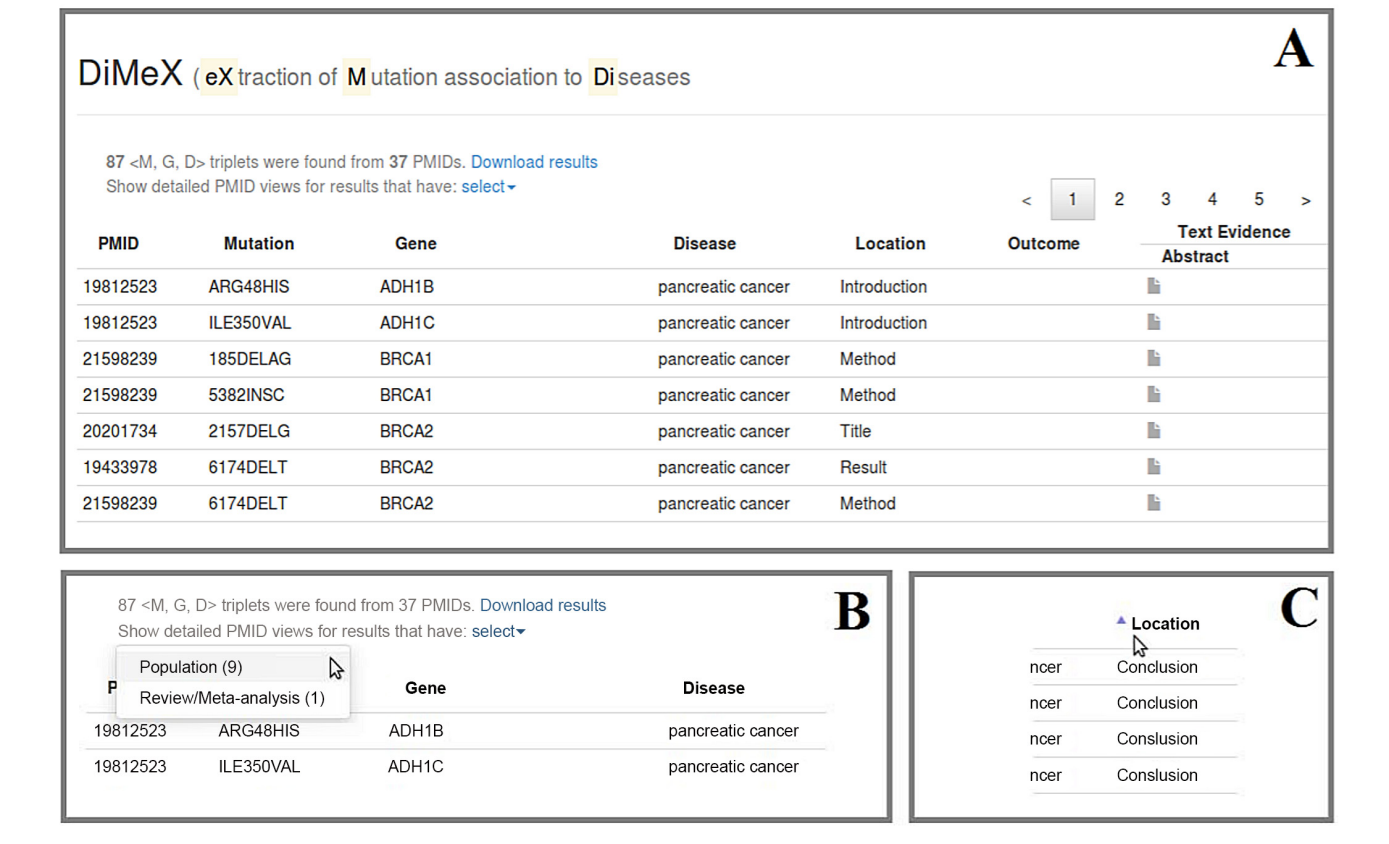
Querying the database with “pancreatic cancer”. (A) Screenshot from the DiMeX website showing a portion of the triplet-view results for the query (B) Options to select PMID-view for abstracts that are review and/or meta-analysis studies or associated with one or more populations. (C) An example showing that the results can be instantly sorted by clicking on the column name.

**Fig 3:**
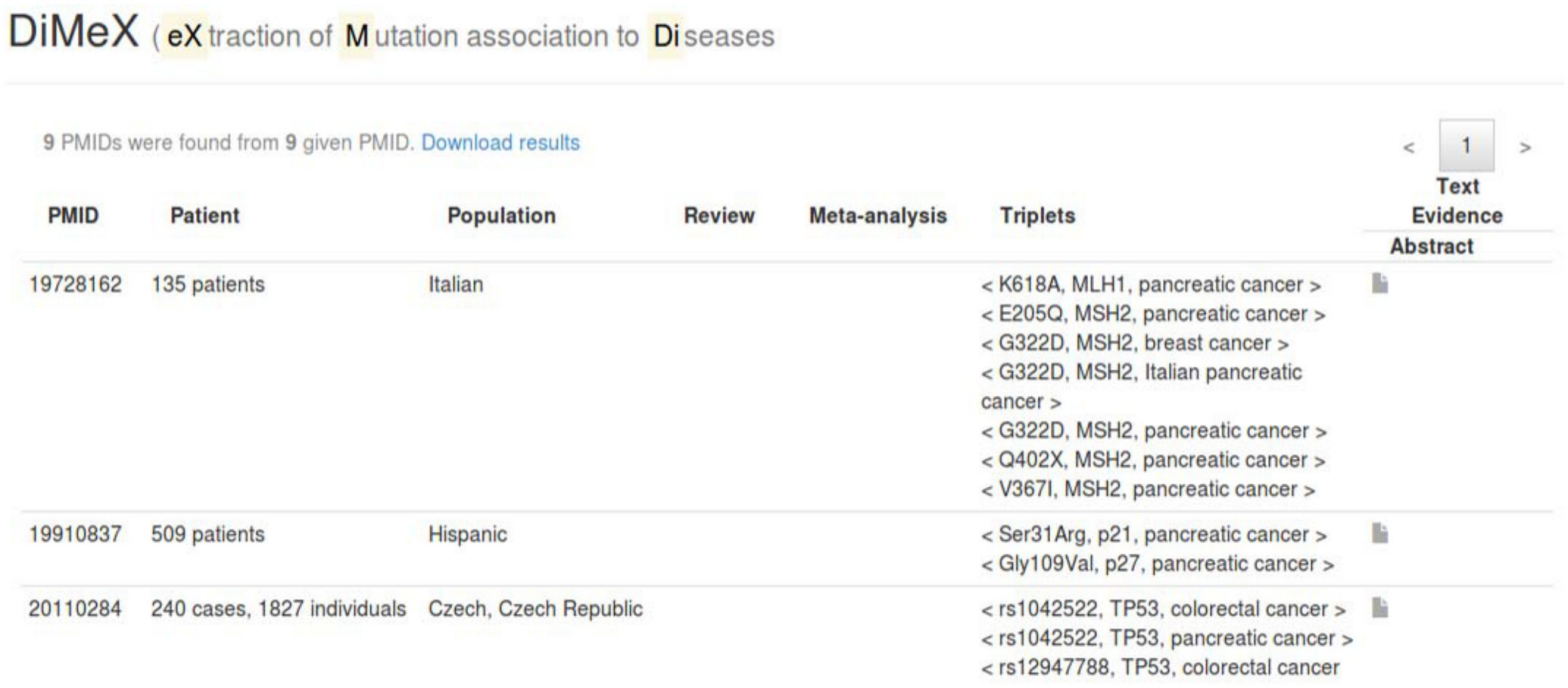
Querying the database with “pancreatic cancer”. This screenshot from the DiMeX website shows a portion of the PMID-view results for abstracts that are associated with one or more populations.

In order to extract the vast amount of mutation-disease association information already available in free text, DiMeX can be applied on the entire Medline collection and store the extracted results in a database. However, we still need to develop a full-functional and user friendly interface to support various types of searches, an undertaking beyond the scope of this work. In the meantime, for illustrative purposes, we make available a database that includes information extracted from roughly 10,000 abstracts and have developed a rudimentary interface. To obtain these abstracts, we ran a search on PubMed using the query *“cancer[tiab]) AND (mutation[tiab] OR variant[tiab] OR polymorphism[tiab])”* and selected abstracts from 2009 to 2011. This yielded a total of 9873 PMIDs, among which 9727 had abstract text. We applied DiMeX on this set of 9727 abstracts, extracting the mutations, associating them with genes and diseases, and storing the triplets <M,G,D> along with the additional information in the local database. The purpose of this exercise was to test the scalability and the ability to support various use case scenarios including those previously mentioned in the discussion of Module C. The results are displayed in two different ways: the triplet-view and the PMID-view. The triplet-view (see Fig 2) shows the extracted triplets and additional information for these triplets. The PMID-view (see Fig 3) shows the information at an abstract level rather than for individual triplets.

Table 7 lists some of the key characteristics of the extractions from the 9727 PMIDs. It is noteworthy that triplets were extracted from only 26% of the abstracts. This is due to the fact that, often, the abstract hints to a possible mutation-disease association but the specific details are contained in the full-length article. 7175 triplets were found across all abstracts, among which 6410 were unique. To evaluate the quality of this large scale extraction of triplets, we have randomly chosen 200 abstracts from the database. Using the same evaluation guideline as in *BiomutaC* dataset, the precision, recall and F-measure was found to be 0.87, 0.86 and 0.87, respectively which is similar to results presented in Table 3. Analysis of the FPs and FNs revealed that most errors were due to gene mentions that were missed by the gene mention detector. If the correct gene for a mutation was missed by the detector, it resulted in an FN. Since our algorithm always attempts to associate a mutation with some gene, the mutations in these cases were associated with some other gene in the abstract, thus yielding an FP as well. Most of the remaining errors were mistakes in picking the wrong disease to be associated with the mutation. The extractions for this set of 200 abstracts are available as supplementary information (S3 Dataset).

**Table 7:** Characteristics of the extracted results. The query used to select the PMIDs is “*cancer[tiab]) AND (mutation[tiab] OR variant[tiab] OR polymorphism[tiab])*”, with abstracts selected from 2009 to 2011.

*Characteristics*	*Counts*
Abstracts	9727
Abstracts with at least one triplet	2511
Total <M,G,D> triplets	7175
Unique <M,G,D> triplets	6410
Unique mutations	3204

Currently, the stored results and the web interface that supports various types of searches can be found at: http://biotm.cis.udel.edu/dimex/. We have conducted the following 3 sample queries into our database to demonstrate the usability of the system.

1.
  - Search the database for the disease *pancreatic cancer*. A search for *pancreatic cancer* retrieved 87 <M,G,D> triplets spread across 37 abstracts from the three year period. Fig 2-A shows a screenshot of the displayed results (partial). The PMID, mutation, gene, disease, extraction zone and related outcome are shown in a tabular format, with the last column (text evidence) providing a link to see the actual abstract text. The entire results table can be downloaded as a spreadsheet with the *Download results* link placed on top of the webpage. The next line shows a link with a select option which opens a pop-up window showing the number of abstracts from which meta-analysis and population information were extracted.
  - Find the populations that were studied for *pancreatic cancer-mutation* associations. To see the populations that are extracted from the resulting PMIDs, the *Population* link in the pop-up window (see Fig 2-B) is clicked. The link switches to PMID-view to display the abstracts that are found to be associated with one or more populations. The population information is associated at the abstract level rather than with triplets, and hence the population information is shown in a separate PMID-view. As seen from Fig 2B, in the case of *pancreatic cancer-mutation* associations, 9 abstracts were connected to one or more populations. Fig 3 displays a screenshot of the PMID-view, which lists the populations for each PMID. For *pancreatic cancer*, the populations were spread across Italian, Hispanic, Czech, German, European, African and Chinese.
2.
  - Search the database for the mutations of MLH1. A search for *MLH1* yielded 96 <M,G,D> triplets spread across 29 abstract, with 59 unique mutations. The results are displayed in the triplet-view.
  - Find the articles that describe studies on diseases affected by the MLH1 mutations. A click on the column name *Location* (as shown in Fig 2-C) sorts and groups together the rows based on the rhetorical zone in the abstract from where the triplets were extracted. According to our hypothesis, articles that describe studies are likely to mention the mutation-disease associations in the Results or Conclusion part of the abstract. By sorting on the column *Location*, it is easy to see that 71 out of the 96 <M,G,D> triplets were extracted from either the Results or Conclusion sections from 23 abstracts. This indicates that these 23 articles describe studies on diseases associated with MLH1 mutations. By sorting on the disease column, we can see that 31 triplets were associated with colorectal cancer, 16 with hereditary non-polyposis colorectal cancer (HNPCC), 11 with gastric cancer, 5 with colon cancer, 4 with prostate cancer, 2 with lung cancer, 1 with pancreatic cancer and 1 with breast cancer.
3.
  - Search the database for the mutation Arg194Trp of XRCC1. A search for XRCC1 Arg194Trp mutation retrieved 35 <M,G,D> triplets spread across 29 abstracts displayed with the triplet-view.
  - Find the meta-analysis studies that have discussed the XRCC1 Arg194Trp mutation. The *Review/Meta-analysis* link in the pop-up window (as shown in Fig 2-B) is clicked to see the 5 abstracts that were detected as meta-analysis or review studies. The PMID-view displays the number of studies being examined for each meta-analysis. In these 5 abstracts, Arg194Trp was associated with oral cancer, esophageal cancer, gastric cancer, colorectal cancer and skin cancer.

## Conclusions

In this paper, we have described the text-mining system DiMeX, which extracts mutations and identifies their association with diseases in Medline abstracts. We have employed NLP techniques to capture the relationship from text. The system achieved state-of-the-art performances for all three tasks, namely mutation detection, mutation-gene and mutation-disease associations. The evaluation results on three different test sets showed that our system outperforms the EMU system. The two separate modules for mutation detection and mutation-gene association are portable and they can be used in the context of any other problem definition, irrespective of the mutation-disease association.

The scalability and robustness of DiMeX were validated by applying it to extract mutation-disease associations from a large set of abstracts in Medline. The extracted mutation-disease associations are stored together with the sentences and the abstracts from which they were extracted, as well as with additional relevant information that were obtained from these abstracts. At this point in time, the website (http://biotm.cis.udel.edu/dimex/) shows a rudimentary interface to a database based on roughly 10,000 abstracts. In order to make the full-fledged database available to the larger scientific community, we will need to work further on the development of the database as well as the search interface. Currently, only the extracted mutations are normalized to a standard format. In a similar way, rather than storing the disease terms appearing in text, we need to standardize them. Normalizing the disease terms to Disease Ontology (DO) terms and IDs will allow for more effective searches as well as enable the use of DO’s hierarchical structure. Similarly, the genes will be normalized to UniProt or Entrez gene IDs. Such developments are beyond the text mining work described here. Finally, we would like to extend our system to run on full-length articles.

## Supporting Information

S1 Dataset *BiomutaC* dataset used for evaluation of DiMeX. (XLSX)

S2 Dataset PMIDs of datasets *PCa_filtered_UD* and *BCa_filtered_UD*. (XLSX)

S3 Dataset Extractions from 200 abstracts used for evaluation of the database. (XLSX)

S1 File List of patterns used to extract mutation to disease association. (TXT)

S2 File List of patterns to detect disease related outcomes and extracted phrases. (TXT)
